# Acupuncture for chronic uncomplicated musculoskeletal pain associated with the spine: A systematic review protocol

**DOI:** 10.1097/MD.0000000000014055

**Published:** 2019-01-11

**Authors:** Tao Xu, Siyuan Zhou, Yutong Zhang, Yang Yu, Xiang Li, Jiao Chen, Jiarong Du, Ziwen Wang, Ling Zhao

**Affiliations:** College of Acupuncture and Moxibustion and Tuina, Chengdu University of Traditional Chinese Medicine, China.

**Keywords:** acupuncture, chronic pain, spine, systematic review

## Abstract

**Background:**

Chronic uncomplicated neck pain, back pain, and lower back pain, with incidences of 18%, 17.7% and 36%, respectively. Although these three conditions occur in different parts of the body, we can summarize them as chronic uncomplicated musculoskeletal pain associated with the spine (CMPS) in accordance with the pathogenesis. Acupuncture is often used to treat them. We aim to conduct a systematic review to evaluate the efficacy of acupuncture for patients experiencing CMPS.

**Methods:**

The following electronic databases will be searched from inception to Mar 2019: Cochrane Central Register of Controlled Trials, Web of Science, ScienceDirect, PubMed, MEDLINE, EMBASE, Springer, WHO International Clinical Trials Registry Platform, China National Knowledge Infrastructure, Chinese Biomedical Literature Database, VIP Chinese Science and Technology Periodical Database, and Wanfang Database. All randomized controlled trials published in English or Chinese related to acupuncture for CMPS will be included. The primary outcome will be the visual analog scale. Adverse events will be evaluated as secondary outcomes for safety evaluation. Study selection, data extraction, and assessment of study quality will be performed independently by two reviewers. RevMan V.5.3.5 software will be used for the assessment of risk of bias and data synthesis.

**Results:**

This study will provide a high-quality synthesis of current evidence of acupuncture for CMPS from visual analog scale.

**Conclusion:**

The conclusion of our study will provide an evidence to judge whether acupuncture is an effective intervention for patients suffered from CMPS.

**Ethics and dissemination:**

Formal ethical approval is not required, as the data are not individualized. The findings of this systematic review will be disseminated in a peer-reviewed publication and/or presented at relevant conferences.

**PROSPERO registration number:**

CRD42018114806.

## 1 Introduction

Three of the most common musculoskeletal conditions associated with the spine (cervical, thoracic, and lumbar) are chronic uncomplicated neck pain, back pain, and lower back pain, with respective incidences of 18%, 17.7%, and 36%.^[1–3]^ These three conditions negatively influence patient quality of life, producing substantial economic and social burdens.^[4,5]^ Epidemiological data demonstrate a strong correlation between neck pain and lower back pain, which can be considered as one condition.^[6]^ Although these three conditions occur in different parts of the body, we can summarize them as chronic uncomplicated musculoskeletal pain associated with the spine (CMPS) in accordance with the pathogenesis. CMPS is treated concurrently with drugs, physical therapy, patient education, and other interventions, but the treatment effect is unsatisfactory.^[7–11]^

Acupuncture is widely used in China and western countries as a complementary and alternative therapeutic technique in various diseases. The effectiveness of acupuncture for treating many diseases has been demonstrated in a series of high-quality clinical trials.^[12–15]^ However, previous research has also found that acupuncture is ineffective in treating some diseases, including fibromyalgia,^[16]^ knee osteoarthritis,^[17]^ and rheumatoid arthritis.^[18]^ An increasing number of clinical studies have reported that acupuncture is effective for CMPS, including chronic neck pain,^[19]^ back pain,^[20]^ and lower back pain.^[21]^ Generally, acupuncture has very few adverse effects, and is thus considered safe.

Although the mechanisms of acupuncture are not yet clear, the biological basis of acupuncture analgesia has been documented in laboratory evidence.^[22,23]^ Acupuncture relief of chronic nonspecific neck pain may be attributed to the inhibition of the excitability of the alpha motor neuron.^[24]^ For lower back pain, acupuncture may influence the blood flow in the sciatic nerve, nerve root, and cauda equina region.^[25]^

There have been many systematic reviews of acupuncture for single chronic neck pain,^[26,27]^ back pain,^[28]^ and lower back pain.^[29,30]^ In the narrow sense, chronic uncomplicated neck pain, back pain, and lower back pain are all examples of skeletal muscle pain associated with the spine. To the best of our knowledge, no systematic review has evaluated the effectiveness of acupuncture for combined neck pain, back pain, and lower back pain. Hence, a comprehensive review is needed to determine whether acupuncture is an effective and safe treatment for CMPS. Herein, we present the protocol for a systematic review that aims to evaluate the effectiveness and safety of acupuncture therapy for patients with CMPS.

## 2 Methods and analysis

### 2.1 Study registration

The protocol for this systematic review was registered with PROSPERO 2018 (registration number: CRD42018114806). This protocol report is based on the Preferred Reporting Items for Systematic Reviews and Meta-Analyses Protocols (PRISMA-P) guidelines.^[31]^ The review will be conducted in accordance with the PRISMA guidelines.^[32]^

### 2.2 Inclusion criteria for study selection

#### 2.2.1 Type of study

Randomized controlled trials evaluating acupuncture therapy for CMPS will be eligible for inclusion, without restrictions on publication status.

#### 2.2.2 Type of participant

Participants aged 18 years or older with CMPS (including chronic neck pain, back pain, or lower back pain) will be included, regardless of their sex, race, education level, or economic status.

#### 2.2.3 Type of intervention

Acupuncture therapy will include body acupuncture, manual acupuncture, fire needling, plum blossom needling, warm needling, and electroacupuncture. Other stimulation methods such as laser acupuncture, dry needling, transcutaneous electrical nerve stimulation, moxibustion, and cupping will be excluded.

Comparison interventions will include sham acupuncture (sham acupuncture at selected acupoints, sham acupuncture at non-acupoints, needling at inappropriate/inactive acupoints, nonpenetrating sham acupuncture, and pseudo-acupuncture interventions),^[33]^ placebo control, western medicine, no treatment (waiting list control), usual care, and other conventional therapies. Additionally, the review will include trials evaluating acupuncture combined with another treatment compared with other typical treatments alone.

#### 2.2.4 Type of outcome measure

The primary outcome will be evaluated by the visual analog scale (range 0–100, where 0 = no pain, and 100 = the worst possible pain). Adverse events will be measured as secondary outcomes for safety assessment.

### 2.3 Search methods for identification of studies

#### 2.3.1 Data sources

The following electronic databases will be searched from inception to Mar 2019: Cochrane Central Register of Controlled Trials, Web of Science, ScienceDirect, PubMed, MEDLINE, EMBASE, Springer, WHO International Clinical Trials Registry Platform, China National Knowledge Infrastructure, Chinese Biomedical Literature Database, VIP Chinese Science and Technology Periodical Database, and Wanfang Database. All randomized controlled trials published in English or Chinese related to acupuncture for CMPS will be included.

#### 2.3.2 Searching other resources

We will scan the reference lists of retrieved studies to identify other eligible studies. Relevant conference proceedings will also be searched.

#### 2.3.3 Search strategy

The search strategy for PubMed is shown in Table 1. The following keywords will be used: chronic pain (eg, “chronic pain” or “chronic ache” or “chronic sore”); neck pain (eg, “neck pain” or “neck disorder” or “cervicalgia” or “cervical pain” or “posterior cervical pain” or “neckache” or “neck muscles”); back pain (eg, “back pain” or “dorsalgia” or “backache” or “back pain without radiation” or “vertebrogenic pain syndrome”); lower back pain (eg, “low back pain” or “lumbar pain” or “lumbago” or “low back ache” or “recurrent low back pain” or “postural low back pain” or “mechanical low back pain”); acupuncture (eg, “acupuncture” or “acupuncture therapy” or “body acupuncture” or “manual acupuncture” or “electroacupuncture” or “fire needling” or “plum blossom needling”); randomized controlled trial (eg, “randomized controlled trial” or “controlled clinical trial” or “random allocation” or “randomized” or “randomly” or “double-blind method” or “single-blind method” or “clinical trial”). The equivalent search keywords will be used in the Chinese databases.

**Table 1:**
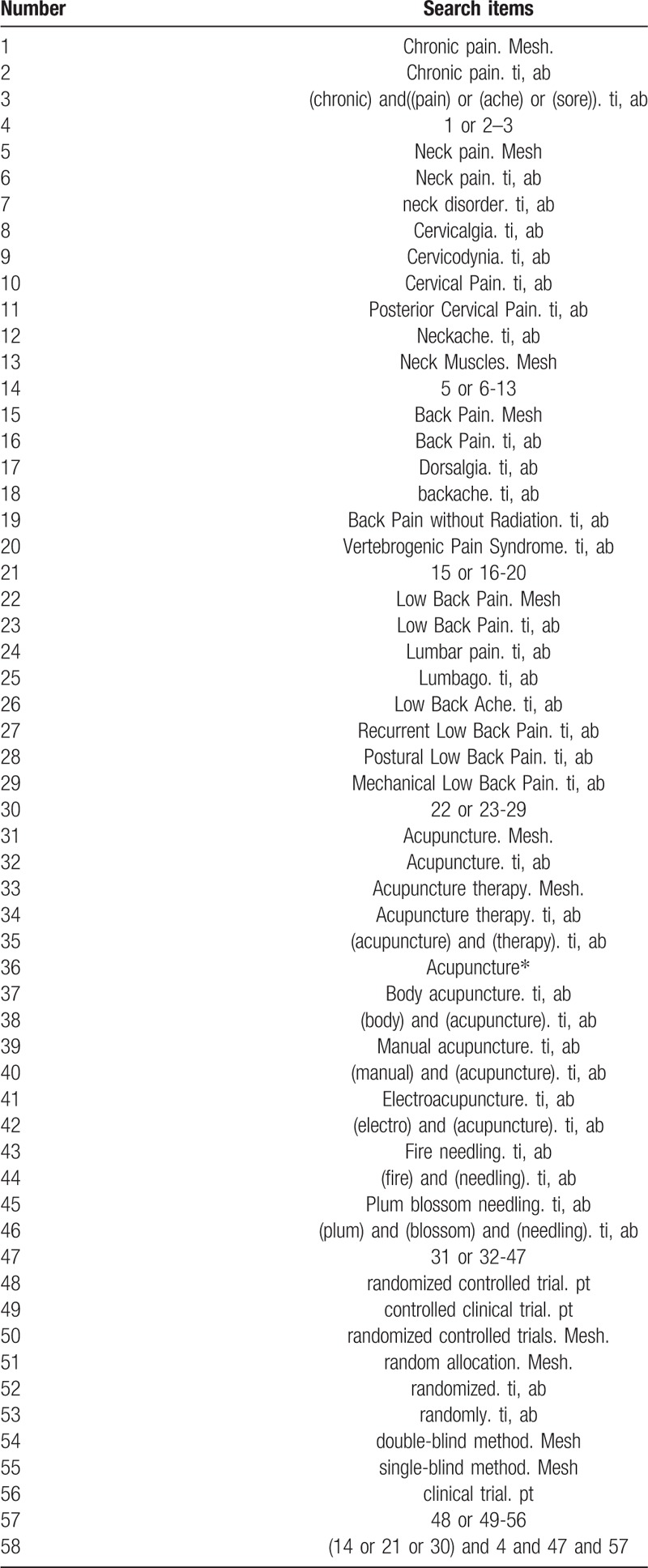
Search strategy for the PubMed database.

### 2.4 Data collection and analysis

#### 2.4.1 Selection of studies

**Figure 1:**
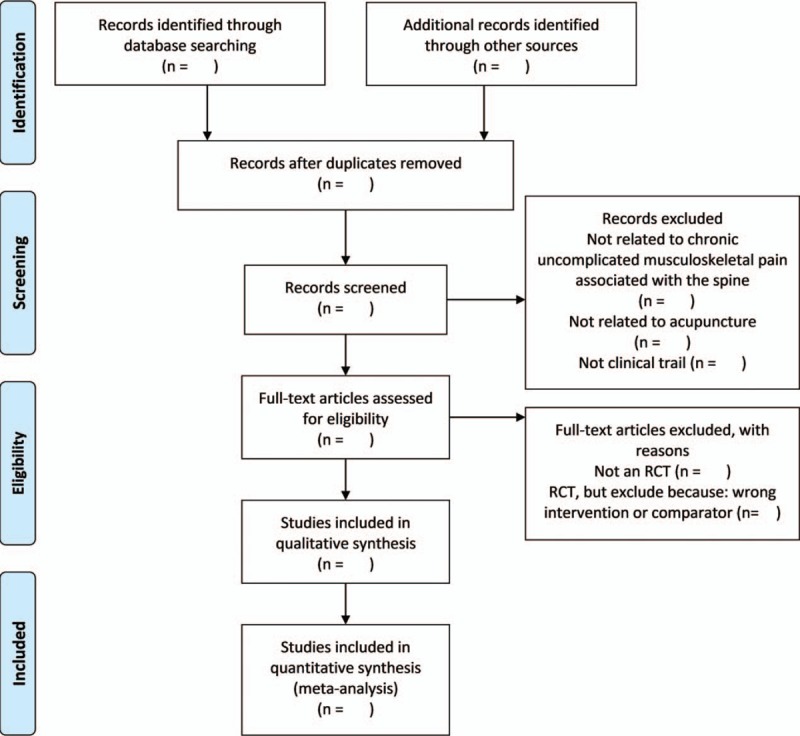
The PRISMA flow chart of the selection process.

Two reviewers will independently review and screen the titles and abstracts of all retrieved studies to identify eligible trials and eliminate duplicated or irrelevant studies in accordance with the inclusion and exclusion criteria; the full text of all potentially eligible studies will then be obtained. Any disagreements will be resolved by discussion with a third reviewer. The study selection process is shown in a PRISMA flow diagram (Fig. 1).

#### 2.4.2 Data extraction and management

The following data will be extracted from the selected studies by 2 independent reviewers using a standard data extraction sheet: year of publication, country, general information, participant characteristics, inclusion and exclusion criteria, sample size, randomization, blinding methods, methods, type of acupuncture interventions, control, outcome measures, results, adverse reactions, conflicts of interest, ethical approval, and other information. Any disagreements will be resolved by discussion with a third reviewer. For publications with insufficient data, we will attempt to obtain the missing data from the authors. All data will be transferred into Review Manager Software (RevMan V.5.3.5) for analysis and synthesis.

#### 2.4.3 Assessment of risk of bias in included studies

For all included studies, 2 reviewers will independently evaluate the risk of bias using the Cochrane Collaboration's tool assessment method, and will complete the Standards for Reporting Interventions in Clinical Trials of Acupuncture (STRICTA) checklist.^[34]^ The risks of bias will be categorized into three levels (low risk, high risk, and unclear) in accordance with the following domains: sequence generation, allocation concealment, blinding of outcome assessors and participants, incomplete outcome data, selective outcome reporting, and other sources of bias. We will attempt to clarify unclear or insufficient items by contacting the corresponding author for more details. Any discrepancies will be resolved by discussion with a third reviewer.

#### 2.4.4 Measures of treatment effect

For dichotomous data, the risk ratio with 95% confidence intervals (CIs) will be used for analysis. For continuous data, the mean difference with 95% CIs will be used. Standardized mean differences with 95% CIs will be used if different scales were used to measure a certain outcome variable. The random-effects model will be used when significant heterogeneity is detected.

#### 2.4.5 Unit of analysis

The analytical unit will be the individual participant.

#### 2.4.6 Management of missing data

The corresponding authors of the included studies will be contacted by reviewers to retrieve any missing or insufficient data of the primary results. If missing data is not available, an intent-to-treat analysis will be performed (including data from all participants in the initial randomly-assigned group), and a sensitivity analysis will be performed to determine whether the results are inconsistent.

#### 2.4.7 Assessment of heterogeneity

We will use the standard χ^2^ test to detect statistical heterogeneity, with the I^2^ test to quantify inconsistency. When the *P* value exceeds .1, and the I^2^ value is less than 50%, studies will be considered homogeneous, and the fixed-effects model will be used. When the *P* value is less than .1, or the I^2^ value exceeds 50%, studies will be considered to have significant statistical heterogeneity, and subgroup analysis will be performed to explore the possible cause; if the heterogeneity remains significant, the random-effects model will be used.

#### 2.4.8 Assessment of reporting biases

If more than 10 studies are included, funnel plots will be used to detect potential reporting biases. The Egger test will be used to determine asymmetry of the funnel plots.

#### 2.4.9 Data synthesis

Data synthesis will be conducted with RevMan V.5.3.5 software. The fixed-effects model will be used for data synthesis if no substantial statistical heterogeneity is detected, while the random-effects model will be used if there is substantial statistical heterogeneity. If there is significant heterogeneity between studies, we will search for possible causes from a clinical and methodological perspective, and provide a descriptive analysis or subgroup analysis.

#### 2.4.10 Subgroup analysis

Subgroup analysis will be performed to explain heterogeneity if possible. Factors such as different types of control interventions and different outcomes will be considered.

#### 2.4.11 Sensitivity analysis

If possible, sensitivity analyses will be conducted to verify the robustness of the review conclusions. The impacts of sample size, study design, methodological quality, and missing data will be evaluated. The analysis will be repeated after the exclusion of studies with low methodological quality.

#### 2.4.12 Grading the quality of evidence

The quality of the evidence will be judged using the Grade of Recommendations Assessment, Development, and Evaluation.^[35]^ The following criteria will be assessed: limitations of the study design, inconsistency of results, imprecision, indirectness, and publication bias. The quality of included studies will be classified into 4 levels: high, moderate, low, or very low.

## 3 Ethics and dissemination

Ethics approval will not be needed because the data that will be used are not individual and no privacy will be involved. The results will be disseminated through peer-reviewed publications or conference presentations. The essential protocol amendments will be documented in the full review.

## 4 Discussion

This systematic review will be the first to assess the effectiveness and safety of acupuncture for CMPS, and its results will address a gap in the literature. The review will be separated into four sections: identification, study inclusion, data extraction, and data synthesis. We believe that this review will aid practitioners in the decision-making process for treating patients with CMPS, and will provide important information for patients and health policy makers.

## Acknowledgments

We thank Dr Kelly Zammit, BVSc, from Liwen Bianji, Edanz Editing China (www.liwenbianji.cn/ac), for editing the English text of a draft of this manuscript.

## Author contributions

TX and SSZ contributed equally to this manuscript and joint first authors. LZ obtained funding. YTZ, YY, JRD and XL drafted the protocol. The search strategy was developed and will be conducted by TX and SYZ. JC and ZWW will obtain copies of the studies and JRD and YTZ will select the studies to be included. TX, SSZ, and JC will extract data from the studies. YY and XL will enter data into RevMan. TX, SSZ, and LZ will conduct the analyses. TX, SYZ, YTZ, and YY will interpret the analyses. TX, SYZ, YTZ, and XL will draft the final review and JC and LZ will update the review. LZ will act as an arbiter in the study selection stage. All authors have read and approved the final manuscript.

**Data curation:** Yutong Zhang, Jiao Chen, Jiarong Du.

**Formal analysis:** Siyuan Zhou, Yutong Zhang.

**Funding acquisition:** Ling Zhao.

**Investigation:** Ziwen Wang.

**Methodology:** Tao Xu, Siyuan Zhou, Ziwen Wang.

**Software:** Yang Yu, Xiang Li, Jiarong Du.

**Supervision:** Jiao Chen.

**Writing – original draft:** Tao Xu, Siyuan Zhou.

**Writing – review & editing:** Ling Zhao.
